# Entropy Analysis of the Flat Tip Leakage Flow with Delayed Detached Eddy Simulation

**DOI:** 10.3390/e21010021

**Published:** 2018-12-28

**Authors:** Hui Li, Xinrong Su, Xin Yuan

**Affiliations:** Key Laboratory for Thermal Science and Power Engineering of Ministry of Education, Department of Energy and Power Engineering, Tsinghua University, Beijing 100084, China

**Keywords:** flat tip, leakage flow, turbulence structures, loss analysis, DDES

## Abstract

In unshrouded turbine rotors, the tip leakage vortices develop and interact with the passage vortices. Such complex leakage flow causes the major loss in the turbine stage. Due to the complex turbulence characteristics of the tip leakage flow, the widely used Reynolds Averaged Navier–Stokes (RANS) approach may fail to accurately predict the multi-scale turbulent flow and the related loss. In order to effectively improve the turbine efficiency, more insights into the loss mechanism are required. In this work, a Delayed Detached Eddy Simulation (DDES) study is conducted to simulate the flow inside a high pressure turbine blade, with emphasis on the tip region. DDES results are in good agreement with the experiment, and the comparison with RANS results verifies the advantages of DDES in resolving detailed flow structures of leakage flow, and also in capturing the complex turbulence characteristics. The snapshot Proper Orthogonal Decomposition (POD) method is used to extract the dominant flow features. The flow structures and the distribution of turbulent kinetic energy reveal the development of leakage flow and its interaction with the secondary flow. Meanwhile, it is found that the separation bubble (SB) is formed in tip clearance. The strong interactions between tip leakage vortex (TLV) and the up passage vortex (UPV) are the main source of unsteady effects which significantly enhance the turbulence intensity. Based on the DDES results, loss analysis of tip leakage flow is conducted based on entropy generation rates. It is found that the viscous dissipation loss is much stronger than heat transfer loss. The largest local loss occurs in the tip clearance, and the interaction between the leakage vortex and up passage vortex promotes the loss generation. The tip leakage flow vortex weakens the strength of up passage vortex, and loss of up passage flow is reduced. Comparing steady and unsteady effects to flow field, we found that unsteady effects of tip leakage flow have a large influence on flow loss distribution which cannot be ignored. To sum up, the current DDES study about the tip leakage flow provides helpful information about the loss generation mechanism and may guide the design of low-loss blade tip.

## 1. Introduction

In a shroudless turbine blade, the pressure difference from the pressure side to the suction side causes a part of the flow from the pressure side passing through the tip clearance to the suction side, thereby the tip leakage flow forms. Because of the large lateral velocity gradient of the leakage flow, it mixes with the secondary flow and causes large loss. The tip leakage flow could account for about 30% of the overall loss in turbine stage [1]. Meanwhile, as the inlet temperature continues to rise, the tip leakage flow could also overheat and damage the blade. Understanding the loss mechanism and developing the loss control method are of great significance for guiding the design of low-loss blade tip and increasing the efficiency of turbine.

Regarding the study of the tip leakage aerothermal performance, there are plenty of mechanism studies of tip leakage flow. Moore and Tilton [2] developed an analytical model to investigate the aerothermal performance of an axial turbine blade, which is based on the potential flow theory. Yaras and Sjolander [3] studied the tip clearance loss by kinetic energy. Tallman and Lakshminarayana [4] studied the effects of tip clearance height and its flow mechanism. Zhou and Zhou [5] proposed a triple-vortices-interaction kinetic model, and a one-dimensional mixing model proposed to explain the vortex interaction. However, the prediction of the mixing loss from the one-dimensional model was lower than Computational Fluid Dynamics (CFD) simulation. With the understanding of the tip leakage flow, there is plenty of experimental and numerical research focusing on the tip geometry. For the cavity tip, Li et al. [6] investigated the effect of cavity depth and the thickness of the squealer rim, and found that the tip leakage flow was enhanced with increasing the thickness of squealer rim. Kang and Less [7] studied experimentally the effects of squealer rim height-to-span ratio on heat/mass transfer rates, and found that, when the squealer rim height-to-span ratio increased, the averaged heat/mass transfer rate on the cavity floor began to decrease steeply and then decreased slowly. For the squealer tip, Yang and Feng [8] studied numerically the tip leakage flow in the first stage rotor blade. They found that the tip gap and groove depth had significant effects on flow and heat transfer. With the groove depth increases to 3% of blade span, leakage flow would be weakened. Senel et al. [9] revealed the influence of squealer width and height on the aerothermal performance of a high pressure turbine blade by numerical calculation. They found that a proper squealer width and height was critical to reduce aerodynamic loss and heat transfer, which is the same as Yang and Feng’s [8]. For the tip with winglet, Joo and Lee [10] employed the naphthalene sublimation technique to investigate the heat/mass transfer characteristics on the winglet top surface for cavity squealer tip and found that the winglet top surface had a lower averaged heat/mass transfer rate than the plane tip with no winglet. In recent years, as the temperature of the gas turbine has increased, the design of the tip cooling method and the cooling structure has also been the research focus. Park et al. [11] measured the heat/mass transfer coefficients and film cooling effectiveness on the tip and inner rim surfaces of blade with a squealer rim, and found that the high film cooling effectiveness was observed in the middle region of tip surface. Ma et al. [12,13] investigated the cooling-base flow interaction in a transonic turbine rotor blade tip. They found that the injection of coolant significantly altered the flow distribution in the tip clearance, and the heat transfer rate is changed by more than 50%. He [14] investigated the heat transfer coefficient and adiabatic film cooling effectiveness on a blade squealer tip with cooling holes. The squealer tip with both tip and pressure-side holes had a higher adiabatic film cooling effect than that with only tip cooling holes.

Since aerodynamics and heat transfer are mutually influenced by each other, there is some research about optimization of blade tips by combining aerodynamics and heat transfer. Caloni and Shahpar [15] applied conjugate analyses to resolve fluid dynamics and thermal distribution for shroudless turbine blade with Thermal Barrier Coating. They found that the tip with opening trailing edge on the suction side provided 0.4% improvement of adiabatic efficiency compared to flat tip. Caloni et al. [16] adopted a multi-objective design optimisation to improve the squealer tip. They found that the combination of leading edge and trailing edge openings showed a significant improvement of aerodynamic performance and the heat load, which compared with a closed squealer tip.

The mechanism study of tip leakage flow is the key point and foundation to understand the flow physics and enhance turbine performance as well as cooling effects. For the unsteadiness of tip leakage flow, there are still many aspects which we need to study further. In order to obtain more insights into turbulence characteristics and the loss mechanism in tip leakage flow, the flow field must be analyzed in more detail. The RANS method is less expensive and serves as the current main simulation method. Du et al. [17] simulated the blade tip by Unsteady Reynolds Averaged Navier–Stokes (URANS) in a high pressure turbine stage. However, with internal flow mechanism study of the turbine, the calculation accuracy of RANS method is not enough to capture the flow details of complex flow, which makes the study of the complex flow mechanism limited. Thus, the high fidelity and accurate numerical simulation is necessary. Kelly et al. [18] adopted the Very Large Eddy Simulation method (a hybrid URANS/LES method) to analyze a squealer tipped axial turbine stage. The VLES results showed a significant improvement to predict the adiabatic efficiency of the turbine stage. That means the hybrid RANS/LES method can obtain a richer flow field structure than RANS. In addition, compared with Large Eddy Simulation (LES) method, the hybrid RANS/LES method is relatively accurate and much cheaper.

In order to further analyze the flow physics, the flow field needs to be decomposed. The POD method is one of the model reduction methods to extract dominant flow structures. The POD method has been widely used in science and engineering, including image processing, data compression, signal analysis, modeling and control of chemical reaction system, turbulence models, and coherent structures. Since Lumley [19] introduced the POD method into turbulence research in 1967, the POD plays an important role in flow analysis. The POD method has been applied in some simple flow studies, such as flat boundary layer flow [20], cylinder flow [21], Couette flow [22], flame combustion [23], turbulence jet in cross flow [24], airfoil flow in wind turbine [25] and so on. However, for some complex flows, such as tip leakage flow, there is a lack of using the POD method to analyze the unsteady turbomachinery flows.

At present, the research on the tip leakage flow mostly adopts RANS or URANS approach, and the in-depth study of tip leakage flow using a high fidelity numerical method is seldom reported. In addition, existing analysis about tip leakage flow focus on the distributions of pressure, temperature and other parameters, while novel analysis methods like POD should provide new perspective about the flow physics. This paper uses the DDES, a hybrid RANS/LES approach to investigate the mechanism of tip leakage flow inside a high pressure turbine blade with a tip gap of 1% height, which has been proved to be superior in capturing a high accuracy and detailed leakage flow. With the POD method, dominant flow modes governing the unsteady evolution in the tip region are successfully obtained. The use of DDES approach as well as POD analysis brings an interesting view of what can be done to better understand the leakage flow with modern methods for computation and post processing. The time-averaged entropy generation rate reveals the overall loss of tip leakage flow, and the instantaneous entropy generation rate shows the local loss. In addition, the unsteady loss obtained by decomposing the loss reveals that the unsteady effects caused by tip leakage flow cannot be neglected.

This paper is organized as follows: firstly, the numerical method and modeling are introduced as follows; secondly, an overview of the computational domain and mesh resolution are given; in the following parts, the detailed numerical results are analyzed; the last section concludes this work.

## 2. Numerical Methods

### 2.1. Governing Equations

The three-dimensional Navier–Stokes equation is expressed as follows:

(1) $$\frac{\partial U}{\partial t}+\frac{\partial F}{\partial x}+\frac{\partial G}{\partial y}+\frac{\partial H}{\partial z}=0.$$

In the above equation, F, G and H represent the fluxes

(2) $$\begin{array}{c} F=\left[\begin{array}{c} \rho u\\ \rho u^{2}+p-\tau_{xx}\\ \rho uv-\tau_{xy}\\ \rho uw-\tau_{xz}\\ \rho uH-\tau_{xx}u-\tau_{xy}v-\tau_{xz}w-\kappa \frac{\partial T}{\partial x} \end{array}\right];G=\left[\begin{array}{c} \rho v\\ \rho uv-\tau_{yx}\\ \rho v^{2}+p-\tau_{yy}\\ \rho vw-\tau_{yz}\\ \rho vH-\tau_{yx}u-\tau_{yy}v-\tau_{yz}w-\kappa \frac{\partial T}{\partial y} \end{array}\right];\\ H=\left[\begin{array}{c} \rho w\\ \rho uw-\tau_{zx}\\ \rho vw-\tau_{zy}\\ \rho w^{2}+p-\tau_{zz}\\ \rho wH-\tau_{zx}u-\tau_{zy}v-\tau_{zz}w-\kappa \frac{\partial T}{\partial z} \end{array}\right], \end{array}$$

where scalar *H* is the total enthalpy, κ is the thermal conductivity, $\tau_{xy},\tau_{yx},\tau_{xz},\tau_{zx},\tau_{yz},\tau_{zy},\tau_{xx},\tau_{yy},\tau_{zz}$ represent the viscous stress tensor component, respectively. $U={\left(\rho ,\rho u,\rho v,\rho w,\rho E\right)}^{T}$ is the conservative variable in the flow field, where ρ is the fluid density, *u*, *v* and *w* represent the velocity components in the Cartesian coordinate system, respectively, and *E* represents the total internal energy ($E=c_{v}T$, $\gamma =c_{p}/c_{v}$, $k=c_{p}\mu /\left(Pr\right)$, μ can be calculated by Sutherland’s law). The state equation p=ρRT is used to close the system of equations, where *R* is the gas constant and *T* is the temperature.

### 2.2. Turbulence Model

The one-equation model, the Spalart–Allmaras model, is simple and has a relatively small amount of calculation. It behaves excellently in wall-bounded flow and is widely used in the aerospace community and the internal flow field. The standard Spalart–Allmaras equation [26] has the form of:

(3) $$\frac{D\tilde{\nu }}{Dt}=\frac{1}{\sigma }\nabla \cdot \left(\nu +\tilde{\nu }\right)\nabla \nu +C_{b1}\tilde{S}\tilde{\nu }-C_{w1}f_{w}{\left(\frac{\tilde{\nu }}{d}\right)}^{2}+\frac{C_{b2}}{\sigma }\nabla \nu \cdot \nabla \nu ,$$

where ν is the kinematic viscosity, and *d* is the distance from the wall surface. $\tilde{S}$ is the production term, defined by

(4) $$\tilde{S}\equiv S+\frac{\tilde{\nu }}{\kappa^{2}d^{2}}f_{v2},\chi =\frac{\tilde{\nu }}{\nu },f_{v2}=1-\frac{\chi }{1+\chi f_{v1}},f_{v1}=\frac{\chi^{3}}{\chi^{3}+C_{v1}^{3}},S=\sqrt{2\Omega_{i,j}\Omega_{i,j}},\Omega_{i,j}=\frac{1}{2}\left(\frac{\partial u_{i}}{\partial x_{j}}-\frac{\partial u_{j}}{\partial x_{i}}\right).$$

The equation for $f_{w}$ is given by

(5) $$f_{w}=g{\left[\frac{1+C_{w3}^{6}}{g^{6}+C_{w3}^{6}}\right]}^{1/6},g=r+C_{w2}\left(r^{6}-r\right),r=\frac{\tilde{\nu }}{\tilde{S}\kappa^{2}d^{2}}$$

and constant parameters used in the turbulence model are

(6) $$\begin{array}{c} \sigma =2/3,\kappa =0.41,C_{b1}=0.1355,C_{b2}=0.622,C_{v1}=7.1,\\ C_{w1}=\frac{C_{b1}}{\kappa^{2}}+\frac{1+C_{b2}}{\sigma },C_{w2}=0.3,C_{w3}=2. \end{array}$$

### 2.3. Delayed Detached Eddy Simulation

In order to overcome the insufficient modeling stress of the Detached Eddy Simulation (DES) method, Spalart et al. [27] updated the DES method to the DDES method, aided by the low-dissipation numerical methods [28,29,30,31]. The DDES method uses a similar equation to the Shear Stress Transport (SST) model proposed by Menter [32] to limit the length scale in the DES method. It can ensure that the switching from RANS mode to LES mode is grid-independent. The DDES method is constructed by modifying the parameter *r* in S-A model as below:

(7) $$r_{d}=\frac{\nu +\nu_{t}}{{\left(U_{i,j}U_{i,j}\right)}^{0.5}\kappa^{2}d^{2}},$$

where $U_{i,j}$ is the velocity gradient. This modification can be applied to any eddy viscosity model. The parameter $r_{d}$ is mainly used to construct the following restriction equation:

(8) $$f_{d}=1-tanh\left({\left[8r_{d}\right]}^{3}\right),\tilde{d}=d-f_{d}max\left(0,d-C_{DES}\Delta \right),$$

where the $C_{DES}$ is 0.65 [33], and Δ is the maximum length scale of mesh element. This equation can effectively limit the “grey area” [27] between the RANS and LES regions, which can overcome the problem of insufficient modeling stress.

### 2.4. Flow Solver

An in-house CFD code, which is based on the Message Passing Interface (MPI)-parallel multi-block structured finite volume method [34] is used in this work. The governing equation of this code is the integral form of the Navier–Stokes equation as below:

(9) $$\frac{\partial }{\partial \tau }\;\int \int \int \;UdV+\frac{\partial }{\partial t}\;\int \int \int \;UdV+\oint \left(F_{c}-F_{v}\right)\cdot ndS=0.$$

The first term on the left side of the equation represents the pseudo time term. In the time marching solution, a three-stage Runge–Kutta/implicit scheme with a multigrid method is used. The second term represents the physical time term, which controls the unsteady evolution. In unsteady calculation, dual-time-step of an optimized, second-order, backward difference temporal scheme (BDF2opt) [35] method is adopted. For high resolution of the small scale strucutres, the ideal numerical method for the convective term should be non-dissipative and discretely conserves the quantities like kinetic energy or entropy; however, this proposes a severe challenge to the stability of the simulation. In this work, to reduce the numerical dissipation, a high-accuracy 5th order Weighted Essentially non-oscillatory (WENO) reconstruction scheme [28,29,36] is adopted and the modified Roe scheme with low Mach number preconditioning [37] is used to compute the convective flux. 4th order centered scheme is used to discretize the viscous term. In the turbulence equation, the source term of the Spalart–Allmaras equation dominates and the discretization methods for the convective and viscous terms have a marginal effect. For stability, a second order upwind method and second order centered scheme are used for the convective and viscous terms in the turbulence model, respectively.

**Figure 1:**
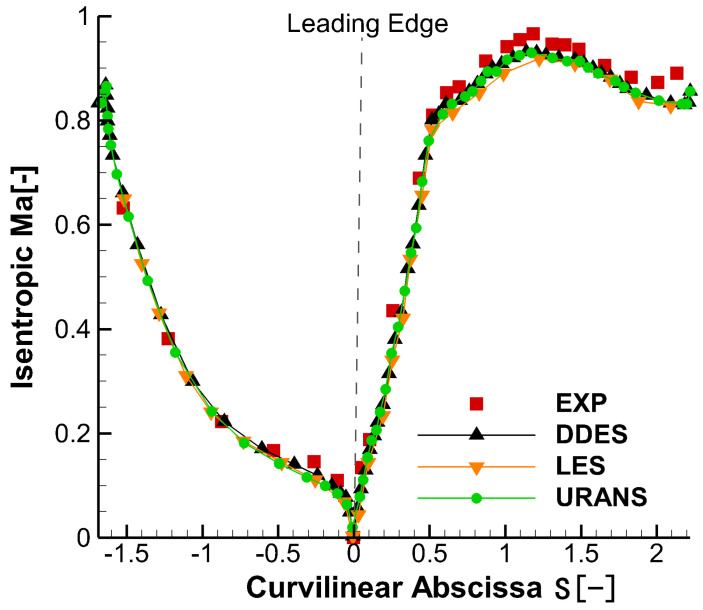
DDES isentropic Ma distributions along the vane surface of case vane [31].

Detailed validation about the DDES method in this code has been conducted by Lin et al. [31] and the same computational approach is used in this paper. For the isentropic Mach number distributions, it is found that the DDES results agree well with the experimental results, as shown in Figure 1.

## 3. Computational Setup

**Figure 2:**
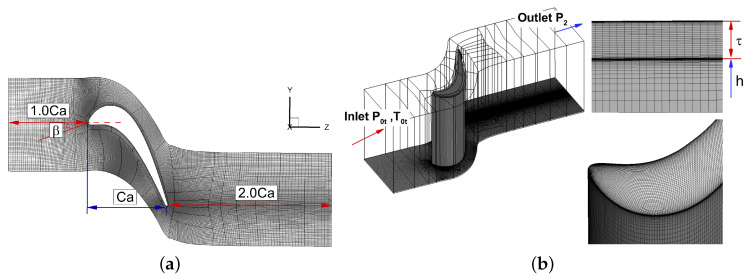
The mesh and blocks distributions. (**a**) Computational domain; (**b**) Boundary conditions and mesh details.

In this paper, the rotor blade in a single stage axial turbine is studied. The relevant parameters of the blade are shown in Table 1. The computational domain is shown in Figure 2a,b, and it is mainly divided into inlet, rotor and outlet blocks. The length of the inlet block is the same as the axial chord length, and the outlet block is twice the length of the axial chord, which can ensure the downstream flow is fully developed.

Commercial software Numeca AutoGrid5 8.9-1 is used to generate multi-block structured mesh. H-Grid topology is applied in general. HO-Grid topology is applied for tip gap. The number of overall mesh points is about 9.11 million, with 53 layers of mesh in the tip clearance and 113 layers spanwise. The minimum orthogonality among all mesh block is 62.1 degrees. The maximum $y^{+}$ of the first layer off-wall mesh is 0.156, which ensures a good resolution of the viscous sublayer. Away from the wall, the mesh element is nearlly isotropic, with $\Delta x^{+}\approx 110$ and $\Delta z^{+}\approx 20$, which are in agreement with the recommended values for LES predictions [38]. In order to facilitate parallel computation, the mesh is split into 58 blocks, with an average of 0.16 million mesh points per block. The periodic boundary condition is set along the pitchwise direction. The surface, tip, shroud and hub of blade are set as no-slip solid surface. The inlet total temperature is 289 K and the inlet total pressure is 100,819 Pa. The outlet pressure is 97,080 Pa. To minimize the numerical reflections at the boundaries, the non-reflecting treatment [39] is adopted at the inlet and outlet surfaces.

## 4. Validation and Comparison of DDES and URANS

**Figure 3:**
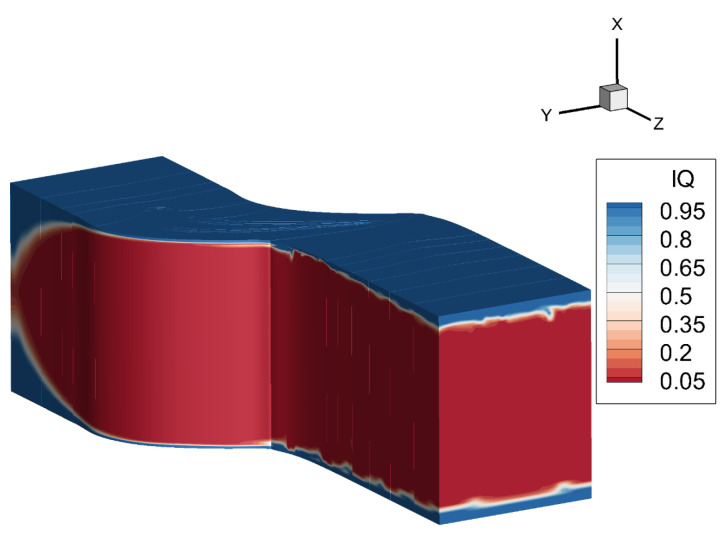
The index IQ distribution.

In the DDES method, sub-grid scale modeling is used away from the wall. The mesh convergence study of DDES is difficult compared to RANS because the modeled part of the turbulence is closely related to the length scale of the mesh element. As a result, refining the off-wall mesh would change the contribution from the subgrid scale (SGS) modeling. Besides checking the distributions of $\delta x^{+}$, $\delta y^{+}$ and $\delta z^{+}$, a useful and widely adopted approach is to check the ratio betwee the resolved and the modeled part of the turbulence kinetic energy. As defined in the following equation, the Index of Quality (IQ) is defined to measure whether the mesh elements resolve the majority of the turbulence kinetic energy [40]:

(10) $$\mathrm{IQ}=\frac{E_{modeled}}{E_{modeled}+E_{resolved}}\approx \frac{C{\left(\frac{\nu_{SGS}}{\Delta }\right)}^{2}}{C{\left(\frac{\nu_{SGS}}{\Delta }\right)}^{2}+\frac{1}{2}\langle {\bar{u}}_{i}^{{}^{\prime }}{\bar{u}}_{i}^{{}^{\prime }}\rangle },$$

where $\nu_{SGS}$ denotes the viscosity defined by the sub-grid scale modeling, and *C*≈ 100 [41], which evaluates the modeled turbulent kinetic energy, $E_{modeled}$. $E_{resolved}$ is the resolved turbulent kinetic energy. Generally, IQ = 0.2 corresponds to the LES needs to resolve more than 80% of the turbulent kinetic energy [40], so that most of the turbulent components can be resolved. It can be seen from Figure 3, IQ for the region away from the wall area is less than 0.2, which indicates the mesh resolution is sufficient and satisfies the LES requirement.

**Figure 4:**
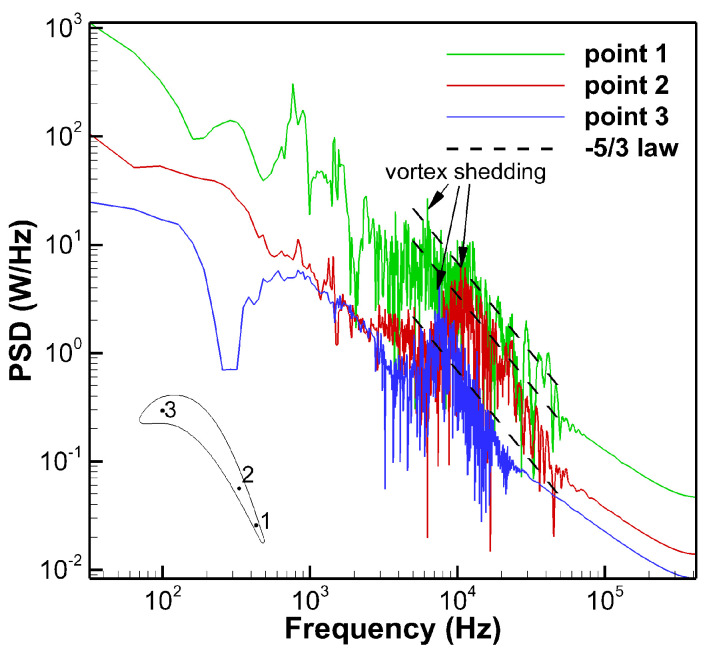
Turbulent velocity power spectral densities at monitoring points.

In the unsteady simulation, the physical time step is taken as 1.2 $\times \;10^{-6}$ s, and 20 pseudo iterations are employed for convergence. After performing Fourier transform on the data captured by the monitoring points, the velocity power spectra is obtained. As shown in Figure 4, an inertial subrange agrees with the Kolmogorov −5/3 law, which indicates that the inertial region of the turbulence is correctly resolved. There are multiple peaks in the power spectral density curve of the same monitoring point, indicating that there are kinds of vortices passing through this monitoring point. In addition, the frequencies of the vortices corresponding to different monitoring points are different.

### Comparison of URANS and DDES Results

**Figure 5:**
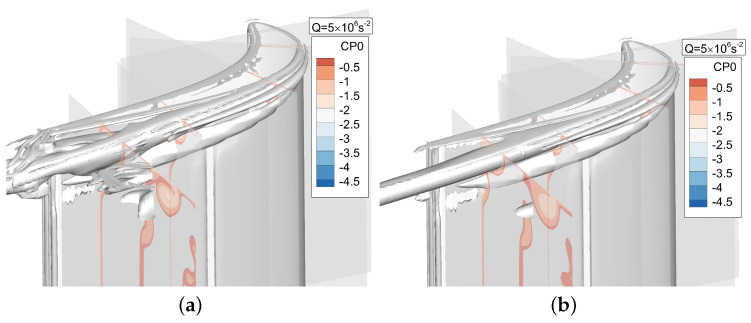
Flow field comparison of DDES and URANS results. (**a**) Instantaneous DDES result; (**b**) Instantaneous URANS result.

Figure 5 shows the results obtained by the DDES method and the URANS method, which are represented by by Q criterion and rendered by total pressure coefficient (Cp0). The Q criterion is defined in the form of: $Q=(\Omega_{ij}\Omega_{ij}-S_{ij}S_{ij})/2,$ where $\Omega_{ij}$ is the vorticity tensor and $S_{ij}$ is the shear strain tensor. The Cp0 is defined in the form of:

(11) $$\mathrm{Cp}0=\frac{P_{0}-P_{0i}}{0.5\rho {U_{m}}^{2}}$$

Both methods capture the tip leakage vortex and up passage vortex. However, the vortex structure of the tip leakage flow and the secondary flow captured by URANS method is basically steady, and the interaction of the two vortex structures is not observed. The DDES method captures the development and interaction process of the tip leakage flow and the secondary flow more precisely, which is very helpful for the analysis of tip leakage flow mechanism in the flow field. It can be seen that the RANS is not suitable for the tip leakage flow, mainly because its failure to predict the interaction between the tip leakage flow and the main flow, which can be traced back to the isotropic and equilibrium assumptions in the currently used linear eddy-viscosity RANS model. In the DDES model, RANS can be considered as the wall-model for the LES simulation. The RANS region is restricted in the boundary layer and, in the current case, the boundary layer is free of separation. As a result, a RANS model like Spallart–Allmaras or SST behaves very well in the boundary layer and performs better than using LES in the whole domain. Thus, the RANS approach is not suitable for the tip leakage flow analysis in the off-wall region.

**Figure 6:**
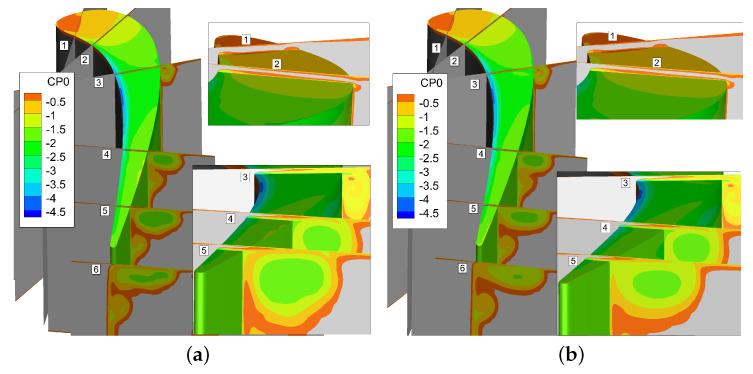
Flow field comparison of DDES and URANS results. (**a**) Instantaneous DDES result; (**b**) Instantaneous URANS result.

**Figure 7:**
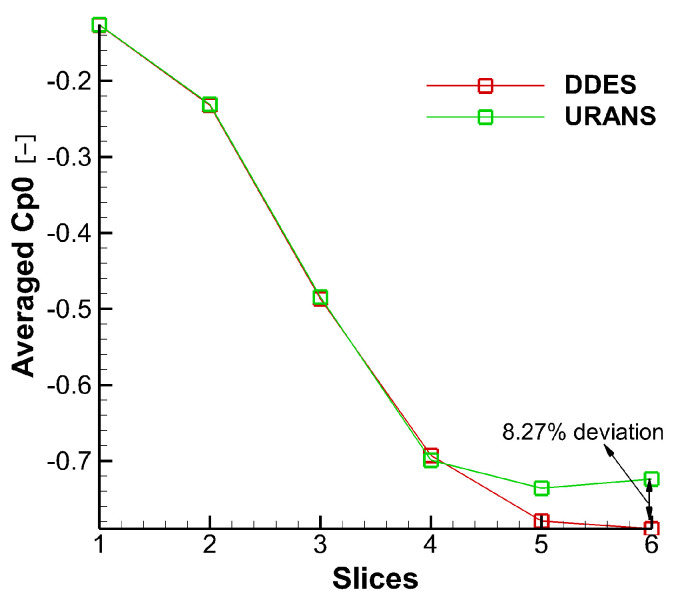
Cp0 comparison of DDES and URANS results.

Instantaneous Cp0 contours are shown in Figure 6. It can be seen from the Cp0 distribution at different slices that URANS results are basically consistent with the DDES results for the first four slices, and the same distribution is also observed in Figure 7.

That is because there is no strong interaction between tip leakage vortex and up passage vortex initially. However, as the tip leakage vortex and the up passage vortex move downstream, the instantaneous results of two models show a clear discrepancy in tip leakage flow. It can be seen that the Cp0 distributions of URANS appear spatially smoothly and nearly no interaction between tip leakage vortex and passage vortex. On the contrary, the DDES method predicts small scale flow structures in tip leakage flow. Since the URANS method does not capture the interaction between tip leakage vortex and up passage vortex, the influence of such interaction on the flow field is neglected. From Figure 7, it can be found that the maximum deviation is about 8.27% between DDES and RANS results. Thereby, URANS underestimates the loss of tip leakage flow.

## 5. Analysis of the Flow Structures

**Figure 8:**
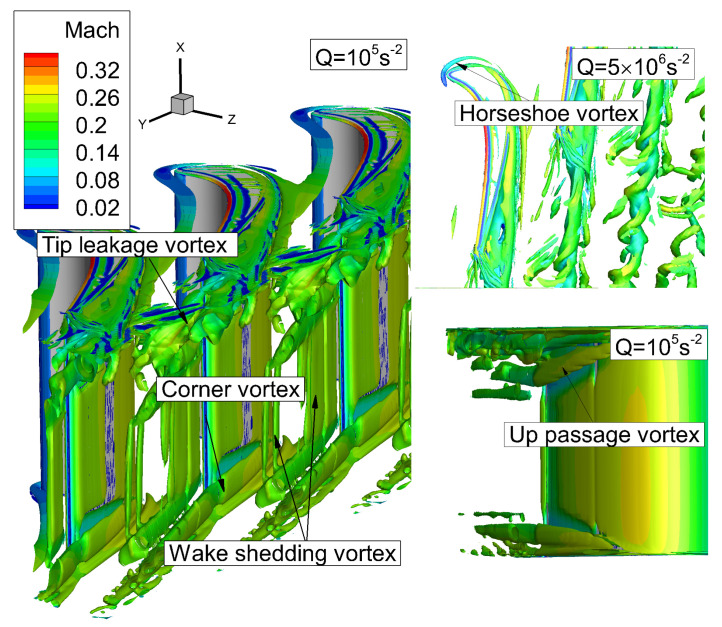
Instantaneous three dimensional vertical structures by Q criterion.

The vortex structures obtained with DDES method are represented by Q criterion and rendered by Mach number, as shown in Figure 8. It has to be noted that, in order to clearly distinguish the vortex structures, different Q criterion values are used to display the flow structures. It can be seen that there is a horseshoe vortex (HV) close to the end wall, and as the horseshoe vortex develops downstream, the boundary layer of the wall surface is continuously entrained. Because of the lateral pressure difference, a passage vortex (PV) is formed and corner vortex (CV) is induced near the blade surface. Similarly, the horseshoe vortex and passage vortex can also be found near the shroud. In order to distinguish the passage vortex near shroud or hub, the passage vortex near shroud is referred to as up passage vortex (UPV). Meanwhile, the wake vortex is also observed at the trailing edge downstream. At the blade tip, due to the pressure difference between the suction side and the pressure side, the fluid flows from the pressure side to the suction side, and then the tip leakage flow is formed. Due to the effect of the tip leakage vortex (TLV), the up passage vortex moves beneath the tip leakage vortex. The tip leakage vortex has stronger turbulence intensity than the up passage vortex. These two structures interact and affect each other. Such multi-scale turbulence structure is difficult to be predicted accurately by the RANS method.

**Figure 9:**
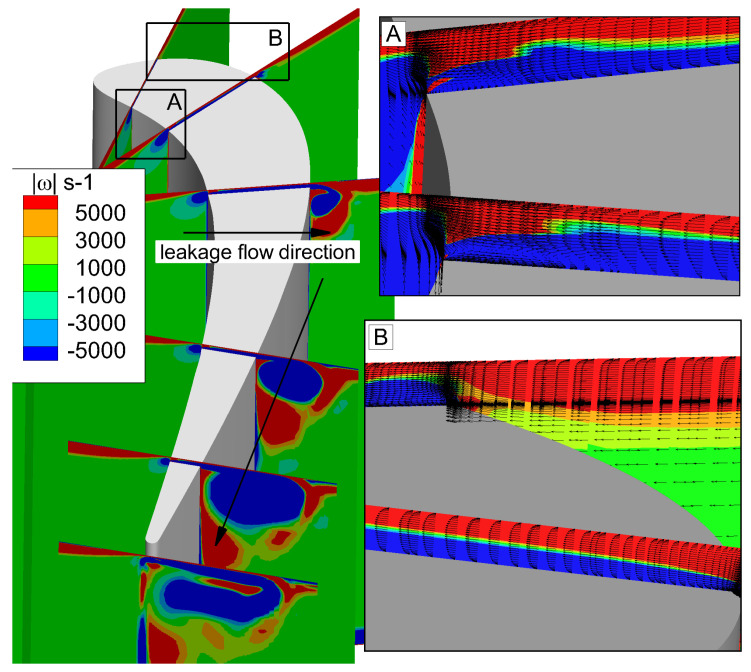
Instantaneous leakage flow process.

**Figure 10:**
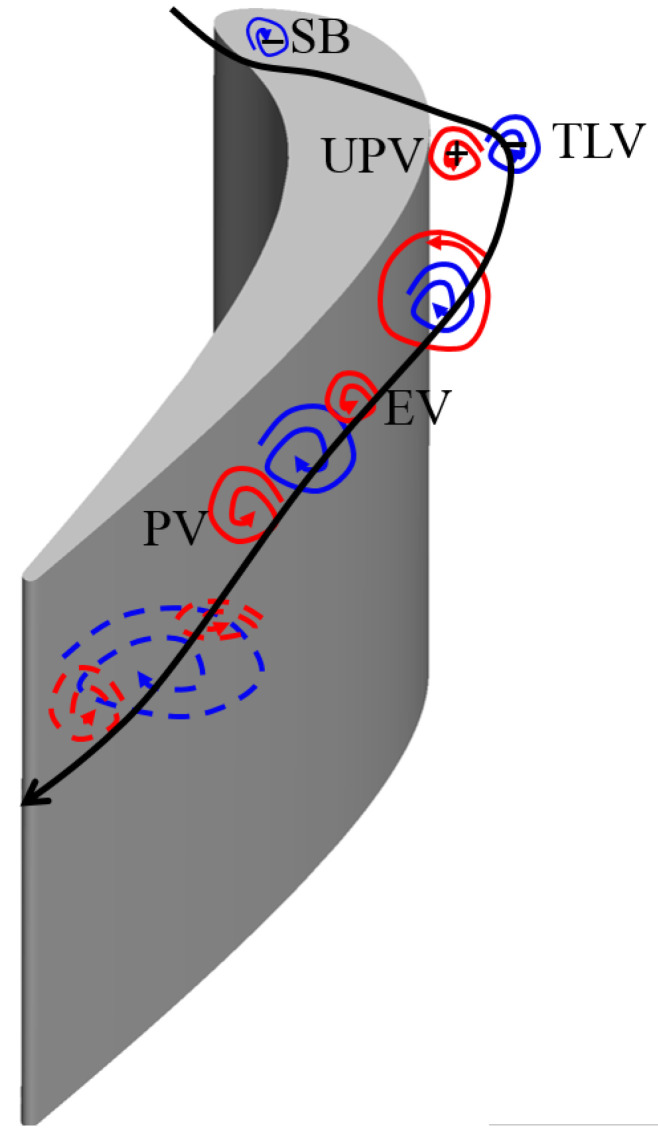
Schematic diagram of tip leakage flow formation and development.

Figure 9 shows the details about the formation and development of the leakage vortex. Six slices are taken along the direction perpendicular to the main flow, which are 10% Ca, 25% Ca, 50% Ca, 75% Ca, 90% Ca, and 100% Ca. Projection of the vorticity vector ∇×u onto the main flow direction u/|u| is displayed on these slices. It shows that at 10% Ca, in the tip clearance, there is separation bubble (SB) near the pressure side. This separation bubble blocks the fluid flows from the pressure side to the suction side. Therefore, there is almost no leakage flow on the suction side. At the same time, the up passage vortex has not yet formed. As moving downstream, the pressure difference between the suction side and the pressure side gradually increases, so that the fluid on the pressure side has more kinetic energy passing through the tip clearance, and then the tip leakage vortex forms at the suction side. Since the energy of the tip vortex is lower in the upstream, the up passage vortex cannot be completely moved by the leakage vortex. As the leakage vortex continues to grow up and develop, the leakage vortex splits the up passage vortex into two parts. One part of vortices is close to the end wall, which is endwall vortex (EV). The second part is below the leakage vortex, which is referred to as passage vortex. As the leakage vortex going downstream, the passage vortex and the leakage vortex gradually merge together. A schematic diagram of the formation and development of tip leakage flow can be used to demonstrate this process, as shown in Figure 10.

**Figure 11:**
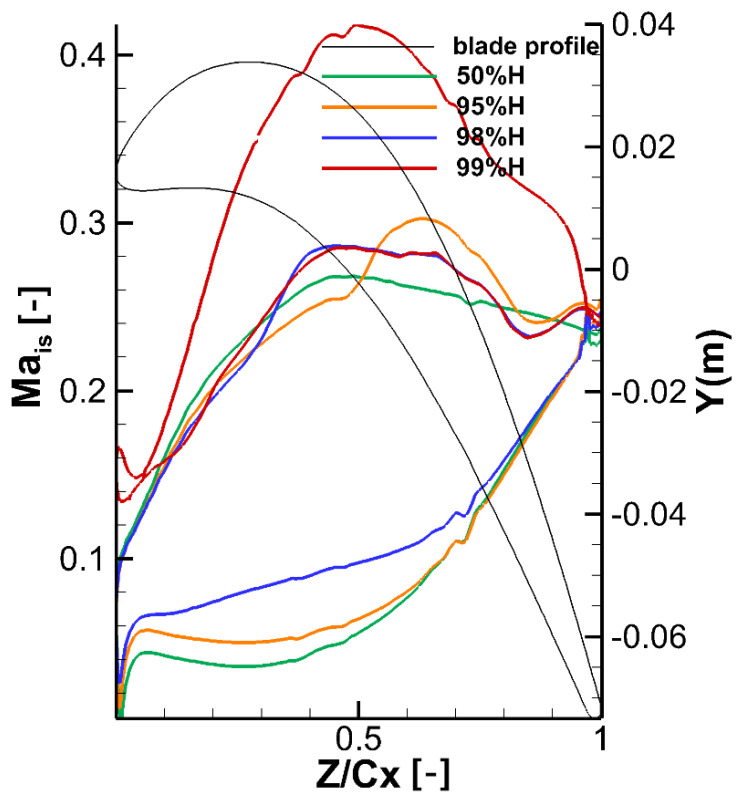
Isentropic Ma distribution along the blade surface.

Different slices are taken along the spanwise direction, and the isentropic Mach number distribution on the blade surface is obtained, as shown in Figure 11. It can be seen that the load on blade surface changes significantly at different slices. At 95% H, before the 55% Ca, the leakage flow goes into a suction side, which increases the pressure on the suction side and decreases the pressure on the pressure side. At 50% H, the isentropic Mach number on the suction side is further reduced while on pressure side the isentropic Mach number increases. On the contrary, the lateral pressure gradient in the passage between adjacent blades decreases pressure on the suction side and increases pressure on the pressure side. In addition, the isentropic Mach number on the suction side further increases and decreases on pressure side. Similarly, at 98% H, due to earlier contact with the tip leakage flow, the isentropic Mach number on the suction side decreases before the 35% Ca, and then the isentropic Mach number increases. On the pressure side, the isentropic Mach number is increased until it is basically the same with the isentropic Mach number of 50% H. Since the velocity of fluid in tip clearance is lower than the mainstream, the isentropic Mach number at 99% H is higher than other slices and the pressure is higher than the mainstream.

**Figure 12:**
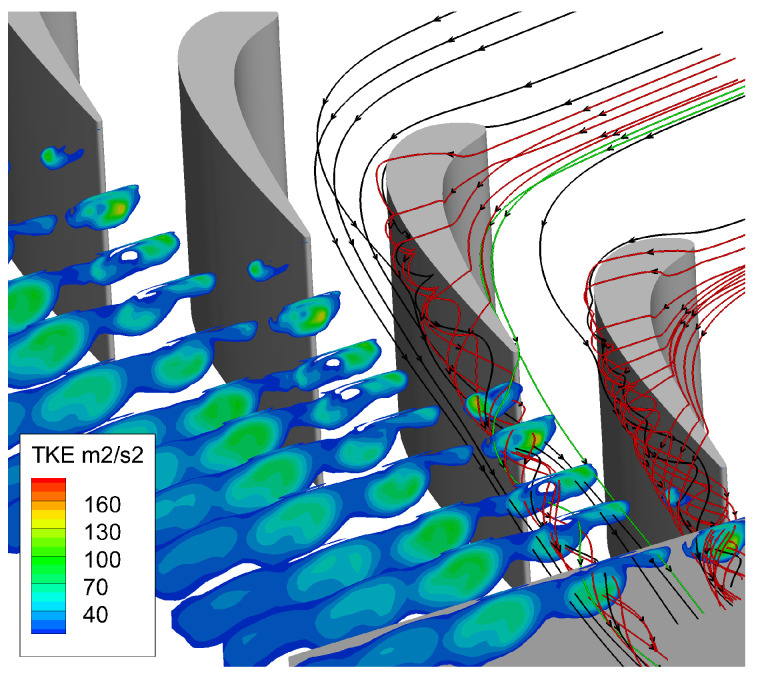
Turbulence kinetic energy distribution.

The distributions of turbulent kinetic energy at several slices along the axial direction are given in Figure 12, where the black solid lines represent streamlines of main flow near the suction side, red solid lines represent the tip leakage flow streamlines, and green solid lines are the streamlines of main flow near the pressure side. The turbulence kinetic energy of the area where the tip leakage flow passes is high, and the turbulence kinetic energy is also high in the place where the passage flow passes. In the process of moving downstream, the intensity of turbulence kinetic energy increases first, and then it gradually diffuses and decreases. This is because the intensity of tip leakage vortex is strong upstream. Then, the interaction between tip leakage vortex and passage vortex causes a part of kinetic energy to dissipate during downstream transportation. It shows that the tip leakage vortex is the main source of turbulent pulsation.

## 6. Modal Analysis and the Loss Mechanism

### 6.1. Proper Orthogonal Decomposition Analysis

POD method, also known as Karhunen–Loeve decomposition or Principle Component Analysis (PCA), is a mathematical tool for analyzing multidimensional data. The employment of POD method in flow field can help identify the main structures and flow characteristics and also obtain modes’ information with different orders [42]. The key of POD solution is to find a set of optimal orthogonal basis $\left\{\varphi_{1},\varphi_{2},\varphi_{3},\ldots ,\varphi_{n}\right\}$ of the function or field space $\left\{v^{n}\left(x\right)\in \Omega \right\}$. $v^{n}\left(x\right)$ is assumed to be approximated by orthogonal bases, so $v^{n}\left(x\right)$ can be represented by the optimal orthogonal basis $\left\{\varphi_{1},\varphi_{2},\varphi_{3},\ldots ,\varphi_{n}\right\}$ as

(12) $$v^{n}\left(x\right)=\sum_{i=1}^{n}a_{i}\varphi_{i}\left(x\right),$$

where $\varphi_{i}\left(x\right)$ is the eigenfunction of $v^{n}\left(x\right)$, $a_{i}$ is POD coefficient and n is number of the eigenfunction. In order to solve $\varphi_{i}\left(x\right)$, the sample of $v^{n}\left(x\right)$ is needed.

It is assumed that there are k linearly independent $v^{n}\left(x\right)$: $v^{1}\left(x\right),v^{2}\left(x\right),\ldots ,v^{k}\left(x\right)$ to form the sample space $V={\left\{v^{i}\left(x\right)\right\}}_{i=1}^{k}$. The dimension is reduced by the optimal method, and the dimension reduction process is equivalent to solving the extreme value problem. That is, the average projection of all elements in set $V={\left\{v^{i}\left(x\right)\right\}}_{i=1}^{k}$ onto the orthogonal basis is maximized as follows:

(13) $$max_{\varphi }\frac{1}{k}\sum_{i=1}^{k}{\left|\langle v^{i},\varphi \rangle \right|}^{2},s.t.{∥\varphi ∥}^{2}=1.$$

The constraint problem in Equation (13) can be transformed into an unconstrained problem by using the Lagrange multiplier method, and then it is treated by variational method. Define

(14) $$K\left(x,x^{{}^{\prime }}\right)=\langle v^{n}\left(x\right),v^{n}\left(x^{{}^{\prime }}\right)\rangle .$$

The primary function $\varphi_{i}\left(x\right)$ needs to meet the following formula:

(15) $$\int_{\Omega }^{}K\left(x,x^{{}^{\prime }}\right)\varphi \left(x^{{}^{\prime }}\right)dx=\lambda \varphi \left(x\right),$$

where $K(x,x^{{}^{\prime }})$ is called kernel function, and is a semi-positive self-correlation matrix. The optimization problem in Equation (13) is transformed into solving eigenvector and eigenvalues of kernel function *K* in Equation (15).

**Figure 13:**
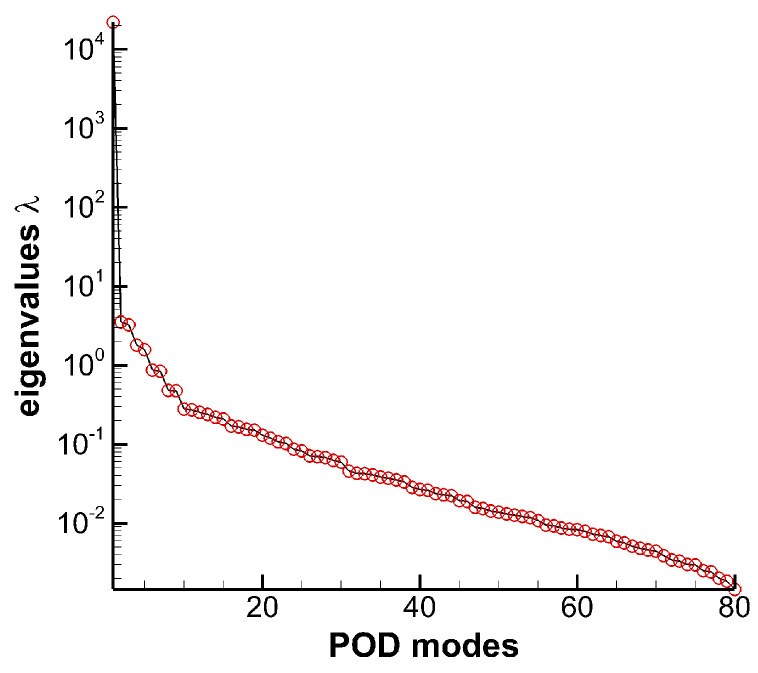
Eigenvalues distribution at different modes.

Conducting POD analysis for the whole flow field would consume too much memory to store the required snapshots, so in the current work the key area of the flow field is extracted which rep the upper half blade span. 80 instantaneous flow fields are collected, which contain about 14 vortex shedding cycles. Eighty modes and its eigenvalues λ are obtained, as shown in Figure 13. Mode 0 represents the time-averaged flow field, and others are the unsteady modes. It can be seen that the eigenvalue decreases rapidly first, then falls slowly after mode 10. Mode 0 occupies a lot of energy, accounting for 99% of total energy. The remaining unsteady flow modes are in pairs. In order to facilitate the analysis, mode 0, mode 1, mode 3, mode 5 and mode 7 are selected for research. In addition, 25 %, 50 %, 75 %, 110% of axial chord slices are extracted.

**Figure 14:**
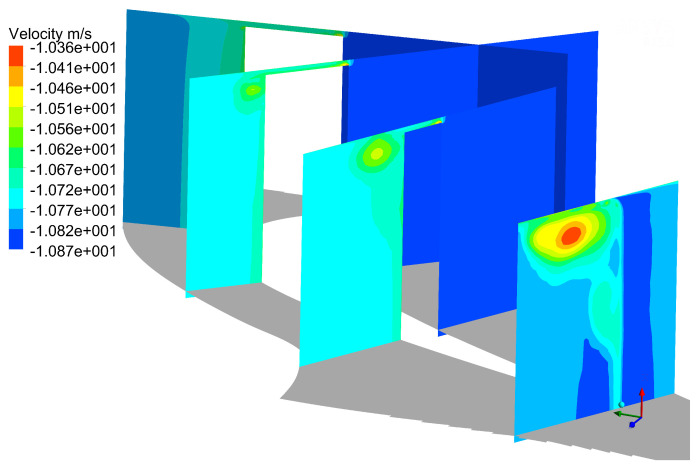
Distribution of POD results at mode 0.

As can be seen, the energy at time averaged mode 0 is the highest, which indicates that most of the flows in the flow field are steady. From Figure 14, the main flow structure is the tip leakage flow vortex and up passage vortex in flow field, and there are no other obvious structures in mode 0. It can be seen that the tip leakage flow vortex gradually becomes larger in the process of moving downstream, and its intensity is stronger than up passage vortex. In time-averaged flow field, the tip leakage vortex is the dominant influencing factor.

**Figure 15:**
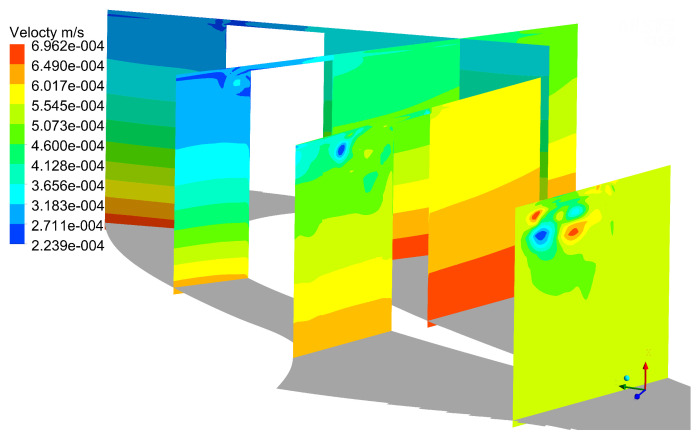
Distribution of POD results at mode 1.

**Figure 16:**
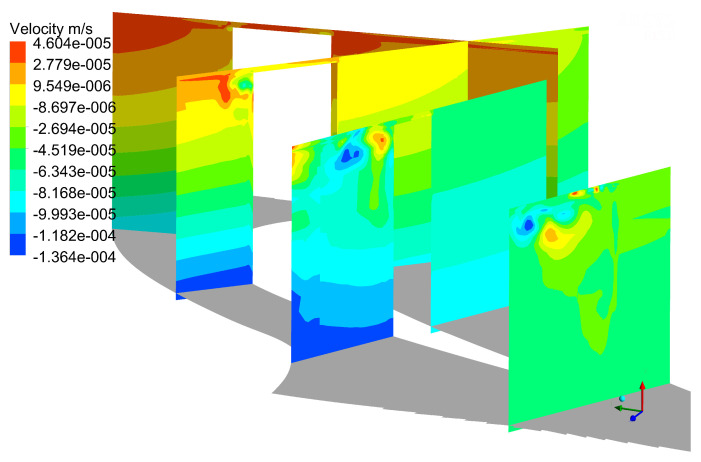
Distribution of POD results at mode 3.

**Figure 17:**
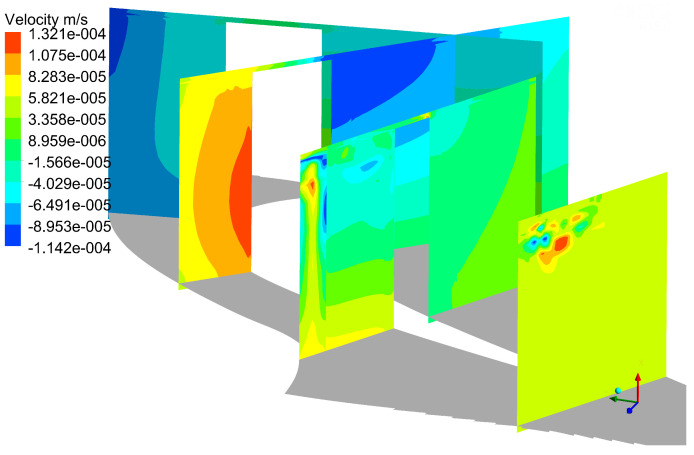
Distribution of POD results at mode 5.

**Figure 18:**
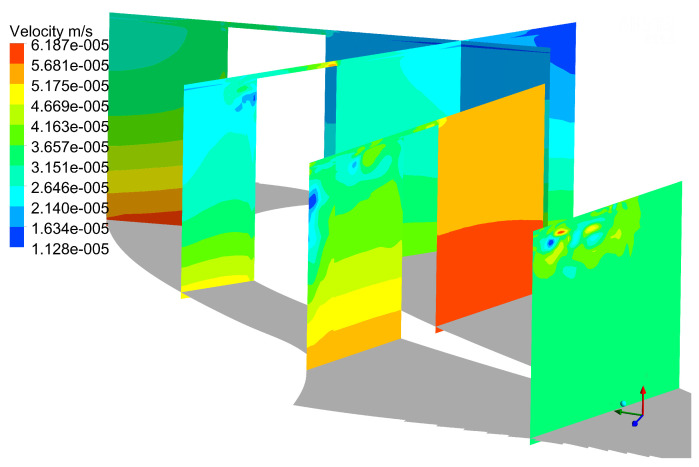
Distribution of POD results at mode 7.

Figure 15 shows the different flow structures compared with mode 0. The unsteady flow structure can be seen on the suction side, and the position of unsteady structure is the same as the tip leakage vortex. It indicates that the tip leakage vortex has unsteady characteristics, and is also the source of unsteady loss in flow field. At 110% of axial chord slice, there are three kinds of vortices as EV, TLV and PV, which is consistent with Figure 10. These unsteady structures are created by interaction between tip leakage vortex and up passage vortex, which reveals that the interaction between the tip leakage vortex and up passage vortex has a strong unsteady flow characteristic. In mode 3, as shown in Figure 16, the quantities of unsteady structures increase in flow field, and its structure dimension gradually decreases. This indicates that the POD method captures small-scale structures in flow field at high order modes. These small structures occupy very lower energy, so it has a less effect on the flow field. As can be seen in mode 5 and mode 7, as shown in Figure 17 and Figure 18, there are no obvious unsteady structures on the suction side at 25%, 50% and 75% of axial slices. However, there is a visible structure in tip clearance, which is caused by the separation bubble in tip clearance. This means that the separation bubble is also the source of unsteady losses in flow field. The unsteady structure on 110% axial chord slice gets smaller, and these small-scale structures contain lower energy, and their turbulence intensity is small.

By using POD, the main unsteady flow structures are obtained, which are tip leakage vortex, up passage vortex, separation bubble, and the vortex generated by interaction of tip leakage and up passage vortex, respectively. Among them, the strongest unsteady characteristic is the tip leakage vortex, which indicates that the tip leakage vortex is the main source of the unsteady effect in flow field.

### 6.2. Loss Analysis

Loss coefficients are generally used to evaluate the flow loss in turbine design, such as energy loss coefficient, enthalpy loss coefficient, and total pressure loss coefficient. Denton [43] studied the enthalpy loss coefficient and total pressure loss coefficient; he found that these loss coefficients were not satisfactory for evaluating the loss of the turbine. He suggested using the entropy loss coefficient as the evaluation index. However, these loss coefficients are only a global value, which is the result of sum of losses, and it cannot provide information about local losses in flow field. The rich numerical simulation results of the flow field now make it possible to measure or visualize the factors which cannot be measured or visualized before. Therefore, the loss coefficient and the loss source can be directly linked together, which is very advantageous for studying the loss mechanism. According to the second law of thermodynamics, the entropy generation rate is a reasonable quantitative measure of irreversibility loss. The entropy generation rate provides detailed information about where the loss occurred, and a direct physical interpretation of the loss can be made by entropy loss, which cannot be provided by loss coefficients. At present, the analysis methods using entropy and entropy generation rate have been applied in basic research such as heat exchanger [44], diffuser [45] and microscopic flow channel [46], etc. Recently, Jin and Herwig [47] used the second law of thermodynamic loss method to analyze the physical mechanism of the influence of wall roughness on turbulence. Lin et al. [31] applied the entropy generation rate to analyze a high pressure turbine. The entropy generation rate is used in this paper to investigate the tip leakage loss.

From the thermodynamics, the irreversibility in the flow field is the source of loss. There are two main types of irreversibility in the flow field: caused by fluid viscosity and heat transfer. The equation is as follows:

(16) $$\frac{\partial \rho s}{\partial t}+\frac{\partial }{\partial x_{j}}\left(\rho U_{j}s\right)=-\frac{1}{T}\frac{\partial Q_{j}}{\partial x_{j}}+\frac{\Phi }{T},\;\mathrm{Sthe}=-\frac{1}{T}\frac{\partial Q_{j}}{\partial x_{j}},\;\mathrm{Svis}=\frac{\Phi }{T}\Phi =\tau_{ij}\varepsilon_{ij}.$$

The left side of the equation is the total entropy generation rate (EGR). The first term on the right side is the irreversible loss caused by heat transfer, defined as Sthe. The second term is the irreversible loss caused by viscous dissipation, defined as Svis.

#### 6.2.1. Distribution of Entropy Generation Rate

**Figure 19:**
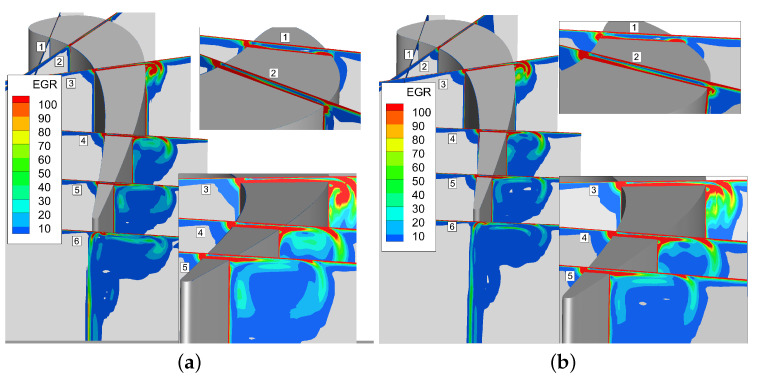
Comparision between normalized total entropy generation rate. (**a**) Instantaneous normalized total entropy generation rate; (**b**) Time-averaged normalized total entropy generation rate.

Figure 19 shows the time-averaged and instantaneous normalized total entropy generation rate, where the EGR value is cut off below 0.5 for displaying the flow loss clearly. It can be seen that the entropy mainly generates in the tip clearance, the area of tip leakage flow vortex, while the up passage vortex and interaction of it and leakage vortex have small entropy generation. It indicates that the tip leakage flow vortex interacts with up passage vortex and weakens the strength of up passage vortex, and then the loss of up passage flow is reduced. There are some differences between instantaneous and time-averaged total entropy generation rate.

**Figure 20:**
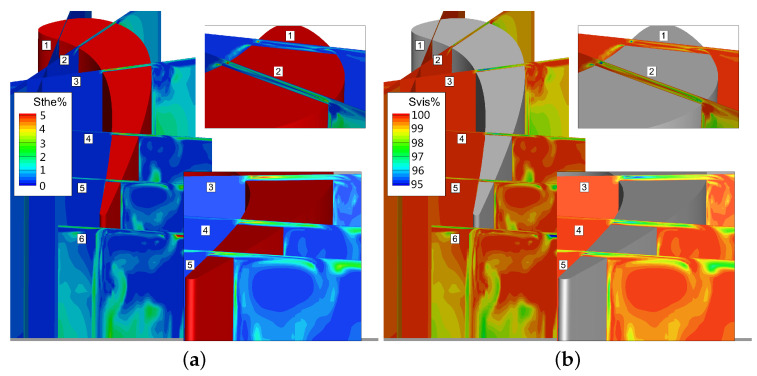
Comparision between normalized entropy generation rate. (**a**) Instantaneous normalized heat transfer entropy generation rate; (**b**) Instantaneous normalized viscous entropy generation rate.

The loss caused by viscous dissipation in this case is much larger than the loss caused by heat transfer, where the Svis is more than 99%, as shown in Figure 20.

It can be seen that the entropy is highest in the tip clearance because of the flow friction dissipation caused by large shear stress of fluid inside the gap. The loss of the tip leakage vortex is greatest at the 50% Ca slice, and then the loss is gradually reduced. The loss in tip leakage vortex core is high due to the strong intensity and high initial energy. As the tip leakage vortex interact with the up passage vortex during the downstream transportation, the tip leakage vortex intensity becomes lower, and the energy in the vortex core is also smaller. Meanwhile, the loss caused by tip leakage flow is lower in the area where the tip leakage vortex and up passage vortex interact with each other.

**Figure 21:**
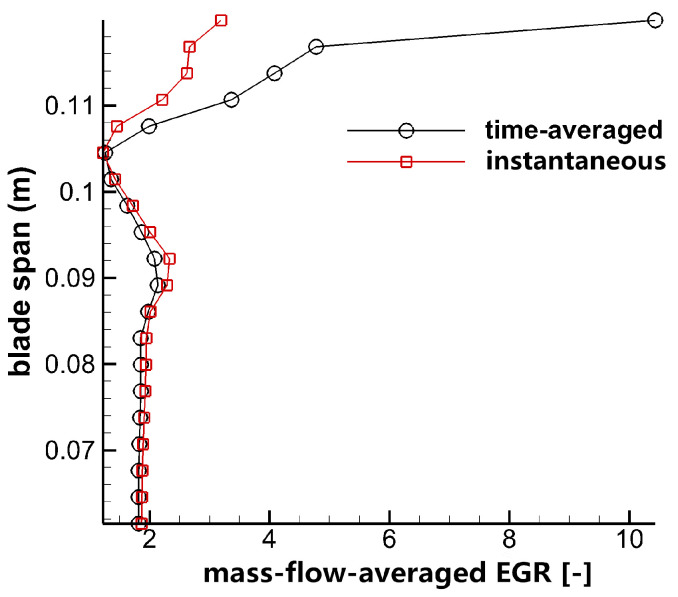
Distribution of mass flow averaged total entropy generation rate.

The mass-flow-averaged entropy generation rate is given at 110% Ca, which is along the upper half span as shown in Figure 21.

There are three large losses in flow field, namely endwall loss, tip leakage flow loss and up passage flow loss. It can be seen that the up passage flow loss is lower than tip leakage flow loss both in both time-averaged and instantaneous results. The difference of up passage flow loss is quite small between time-averaged and instantaneous results. However, the difference of tip leakage flow is very large, which means that the unsteady loss of tip leakage flow is larger than passage flow loss. The time-averaged results of tip leakage flow are larger than instantaneous results; it indicates the turbulence characteristics in tip leakage flow is reduced by interacting with up passage flow and dissipating a large amount of energy in the transportation process. Therefore, if losses are only analyzed by time-averaged flow, the loss evaluation is over-predicted.

#### 6.2.2. Losses Due to Steady and Unsteady Effects

In addition to classifying losses from physical sources, losses caused by the actual flow process can also be composed of two parts: losses due to steady effects (contribution from the time-averaged flow field, $EGR(\bar{U})$) and unsteady effects (instantaneous flow field subtracts time-averaged flow field, ${EGR}^{\prime }=EGR-EGR\left(\bar{U}\right)$). With steady-state simulation using the RANS method, the unsteady evolution of the flow field is not available. For cases with simple flow structure, it yields satisfying loss prediction; however, the steady simulation method based on RANS often ignores or underestimates the influence of unsteady effects on the flow field, when analyzing the losses. The unsteady flow effects enhance the irreversibility of flow field, especially the large-scale periodic unsteady effects. In order to understand this problem more clearly, the steady and unsteady results are selected for further analysis.

**Figure 22:**
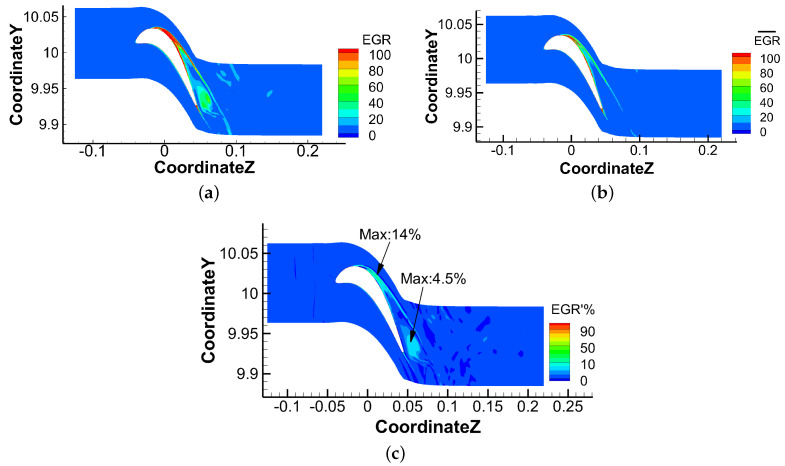
Comparison of losses due to steady and unsteady effects at 98% span. (**a**) Instantaneous EGR; (**b**) EGR of time-averaged flow; (**c**) Losses due to unsteady effects.

Figure 22 shows the instantaneous, steady and unsteady results at 98% of blade span. It can be found that losses in instantaneous field occur in tip leakage flow, up passage flow and wake area, especially caused by the interaction between tip leakage flow and up passage flow. The losses due to unsteady effects show the difference between the instantaneous loss and steady loss. It indicates that the unsteadiness in tip leakage flow along the flow direction cannot be neglected, since the losses caused by unsteadiness can be as high as 14% near the leading edge and 4.5% near the trailing edge on the suction side. These unsteady losses are mainly caused by the unsteadiness of tip leakage flow and its interaction with passage flow.

**Figure 23:**
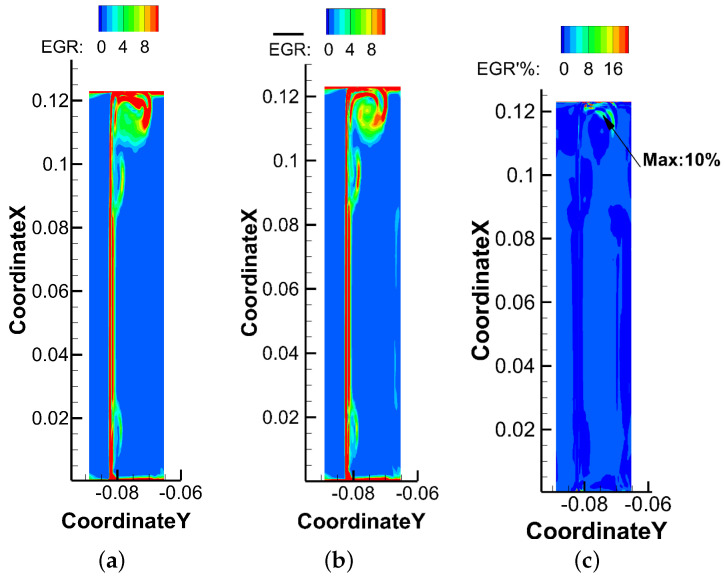
Comparison of losses due to steady and unsteady effects at 110% axial chord. (**a**) Instantaneous EGR; (**b**) EGR of time-averaged flow; (**c**) Losses due to unsteady effects.

Figure 23 displays the losses distribution at 110% of axial chord. The instantaneous results show that losses mainly occur in tip leakage vortex, up passage vortex and wake area. The largest losses take place close to the end wall, and the loss of up passage vortex is smaller than loss of tip leakage vortex. The differences due to steady and unsteady effects appear on the tip leakage vortex, and the maximum loss caused by unsteadiness accounts for 10%, which reveals that the tip leakage flow has strong unsteady characteristics.

It can be seen from the steady and unsteady losses distribution in the flow field that the tip leakage flow has strong unsteady characteristics along flow and spanwise direction. It indicates that the tip leakage flow has large-scale turbulent and three-dimensional flow characteristics. Unsteady losses in tip leakage flow cannot be ignored.

## 7. Conclusions

This work aims to investigate the flow phenomena and loss mechanism of tip leakage flow. The rich and detailed flow fields show the generation and development of the tip leakage flow. In addition, a schematic diagram comes up for explaining the flow field clearly. The flow field is decomposed by the POD method, which helps to resolve the main unsteady structures. The loss obtained by entropy generation rate is helpful to understand the loss caused by tip leakage flow and its interaction.

POD is used to identify the dominant energy containing modes in flow, and it is found that the main flow structures near the shroud are tip leakage vortex and passage vortex in time-averaged flow. Apart from these structures, there is the separation bubble in the unsteady flow field. The strongest unsteady characteristic occurs on the 110% of axial chord slice, where the tip leakage vortex interacts with the up passage vortex. This is the main source of unsteady losses in flow field.

In tip leakage flow, the irreversible loss caused by viscous dissipation is the dominant factor. Meanwhile, the main loss generated in flow field is due to a separation bubble in tip clearance, tip leakage vortex and the end wall vortex formed by up passage vortex. The friction dissipation caused by separation bubble in tip clearance causes the local maximum loss. The loss of the tip leakage vortex has the strongest influence at 50% Ca. As the up passage vortex interacts with the tip leakage vortex, the loss caused by tip leakage flow is reduced in the flow field. In addition, the unsteady effects of tip leakage flow have a large influence on flow losses distribution, which cannot be ignored. 

## Figures and Tables

**Table 1:** Design parameters of the linear turbine cascade.

Parameter	Value
Blade Span, h	123 mm
Blade Axial Chord, Ca	0.69 h
Blade Pitch, p	0.8 h
Tip Gap Height, τ	0.01 h
Relative Inlet Flow Angle, β	20${}^{{}^{\circ}}$
Inlet Reynolds number	230,000

## Abbreviations

The following abbreviations are used in this manuscript:

h	blade span, mm
Ca	blade axial chord, mm
*C*	blade chord, mm
ρ	density, kg/m${}^{3}$
p	blade pitch, mm
*P*	pressure, Pa
$P_{0}$	total pressure, Pa
$P_{0i}$	total pressure at inlet, Pa
$U_{m}$	velocity at mid-span, m
*T*	temperature, K
*s*	entropy, kJ/(kg·K)
|ω|	projection of the vorticity, s${}^{-1}$
Φ	dissipation, kJ/(s·m${}^{3}$)
τ	tip clearance height, mm
β	relative inlet flow angle, °
m	inlet mass flow rate, kg/s
IQ	index of quality
Cp0	total pressure coefficient
TKE	turbulent kinetic energy, m${}^{2}$/s${}^{2}$
EGR	normalized entropy generation rate
Svis	viscous entropy generation rate
Sthe	thermal entropy generation rate
$\tau_{ij}$	viscous stress, kJ/m${}^{3}$
$\varepsilon_{ij}$	deformation, s${}^{-1}$
